# Probing the Interoceptive Network by Listening to Heartbeats: An fMRI Study

**DOI:** 10.1371/journal.pone.0133164

**Published:** 2015-07-23

**Authors:** Nina I. Kleint, Hans-Ulrich Wittchen, Ulrike Lueken

**Affiliations:** 1 Institute of Clinical Psychology and Psychotherapy, Technische Universität Dresden, Dresden, Germany; 2 Neuroimaging Center, Department of Psychology, Technische Universität Dresden, Dresden, Germany; 3 Department of Psychiatry, Psychosomatics, and Psychoatherapy, University Hospital Würzburg, Würzburg, Germany; Laureate Institute for Brain Research and The University of Oklahoma, UNITED STATES

## Abstract

Exposure to cues of homeostatic relevance (i.e. heartbeats) is supposed to increase the allocation of attentional resources towards the cue, due to its importance for self-regulatory, interoceptive processes. This functional magnetic resonance imaging (fMRI) study aimed at determining whether listening to heartbeats is accompanied by activation in brain areas associated with interoception, particularly the insular cortex. Brain activity was measured with fMRI during cue-exposure in 36 subjects while listening to heartbeats vs. sinus tones. Autonomic markers (skin conductance) and subjective measures of state and trait anxiety were assessed. Stimulation with heartbeat sounds triggered activation in brain areas commonly associated with the processing of interoceptive information, including bilateral insular cortices, the inferior frontal operculum, and the middle frontal gyrus. A psychophysiological interaction analysis indicated a functional connectivity between the middle frontal gyrus (seed region) and bilateral insular cortices, the left amygdala and the supplementary motor area. The magnitude of neural activation in the right anterior insular cortex was positively associated with autonomic arousal. The present findings indicate that listening to heartbeats induced activity in areas of the interoception network as well as changes in psychophysiological arousal and subjective emotional experience. As this approach constitutes a promising method for studying interoception in the fMRI environment, a clinical application in anxiety prone populations should be addressed by future studies.

## Introduction

The perception of one’s heartbeat conveys important information for homeostatic and self-regulatory processes which are related to interoception [1–3]. In anxiety sensitive subjects or patients suffering from panic disorder, elevated attention towards cardiovascular processes (e.g. perceiving one’s own heartbeat) serves as a main trigger for catastrophic interpretations that may ultimately result in a panic attack within a vicious circle framework [4]. Experimental approaches to study interoceptive processing use heartbeat perception or detection tasks such as the well-established Schandry task [5, 6]. Neurofunctional substrates of heartbeat perception encompass the insular cortex as a main hub for interoceptive processing and, depending on anxiety sensitivity, partly overlap with fear circuitry structures such as the amygdala [7]. However, detecting one’s own heartbeat as requested by the Schandry task in a noisy functional magnetic resonance imaging (fMRI) environment can be quite challenging. Listening to external auditory heartbeats could represent a promising alternative to study interoceptive processing with high applicability within the fMRI environment. We therefore aimed at probing the neural interoceptive network during the processing of heartbeat sounds.

Interoceptive information is cortically represented in the insular cortex [2, 8]. The insula has been reported to exhibit a functional posterior to anterior gradient [9] with the dorsal posterior insula primary processing interoceptive representations, whereas integration of interoceptive information with higher cognitive and affective components is associated with anterior insular cortex (AIC) functions. Findings from intracranial recordings during electrocortical stimulation [10] substantiate this neurofunctional distinction, showing that somato- and viscerosensory symptoms were exclusively elicited during stimulation of the posterior or central insular lobe. Evidence from fMRI studies further supports the conceptualization of the (anterior) insula as a key structure for interoceptive information processing reported for the cardiovascular [11–13], tactile [14], gastrointestinal [15], and respiratory systems [16, 17], incorporating higher representations and stimulus evaluation, i.e. affective and sensory experience [14]. Adjacent structures to the insula, e.g. the inferior frontal operculum (a transition zone between the insula and frontal operculum), are likewise associated with experiencing one’s own feeling state [18, 19]. The anterior cingulate cortex (ACC) is suggested to play an important role in the integration of interoceptive information and regulation of homeostasis [20] as supported by a strong connectivity of ACC and AIC [21]. Anticipation of interoceptive threat has been shown to activate the AIC, ACC and the dorsomedial prefrontal cortex, possibly reflecting preparation of active avoidance [16]. Tracy et al. [22] argue that the posterior parietal cortex (PPC) is implicated in awareness to internal visceral states, since the PPC is also implemented in an attention brain network for external signals. According to these authors, the PPC is involved in situations demanding internal monitoring of interoceptive signs without external information being present.

The present study aimed at investigating neural activity while listening to heartbeat sounds and their overlap with the above described interoceptive and fear processing network. On the basis of accumulating evidence for an insular-opercular network, amygdala involvement in processing stimuli of biological significance and more adjacent prefrontal regulatory structures mediating interoceptive awareness, sensitivity, or avoidance preparation [2, 8, 16], as well as the PPC in reallocating attentional ressources [12], we expected neurofunctional activation in this network in the heartbeat condition which is supposed to trigger an internal image of interoceptive awareness. Moreover, we expected that particularly accelerated heartbeats would elicit strong activation in the interoception neural network inasmuch as elevated heartbeats, being an indicator of sympathetic arousal in response to internal or external challenges, may trigger the need for homeostatic adaption guided by interoceptive processing. Thus, we introduced two heartbeat conditions to assess effects of stimulus frequency: a resting heartbeat condition with 50 beats per minute (bpm) and an accelerated heartbeat condition with 100 bpm that were contrasted to a sinus tone condition (50 vs. 100 bpm). Based on previous studies associating anxiety proneness with increased insular activation [23], we expected the magnitude of insular activation being positively correlated with autonomic and subjective markers of arousal, as well as with trait markers of anxiety sensitivity. Further testing the functional validity of the task, we expected a positive functional connectivity between neural activation patterns during heartbeat processing and key nodes of the interoceptive and threat processing network.

## Method

### Sample characteristics

Thirty-nine student volunteers (29 women) from the Technische Universität Dresden were recruited via an online-screening procedure implemented at the Neuroimaging Center to volunteer for MRI studies. Subjects were eligible for fMRI assessments, had sufficient (corrected) visual acuity and hearing, were right-handed and reported being free of any lifetime neurological condition, current psychiatric complications, and current psychoactive medication. As n = 3 subjects had to be excluded retrospectively due to structural abnormalities as assessed by an experienced neuroradiologist, a total of n = 36 data sets remained eligible for analyses. Regarding skin conductance (SC) data, n = 1 subject had to be excluded from analysis due to technical failure.

### Procedure and fMRI task

After being familiarized with the task, subjects completed the Edinburgh Handedness Inventory (EHI; [24]) to ensure right-hand-dominance and were then prepared for fMRI assessment; electrodes for SC measurement were attached. Following the fMRI assessment, subjects completed questionnaires assessing anxiety sensitivity (Anxiety Sensitivity Index: ASI; [25]) and depressive symptoms (Beck Depression Inventory II: BDI-II; [26]). Subjects received 10 € for participation. All subjects provided their written informed consent to participate in the study. The ethics committee of the Technische Universität Dresden (Germany) approved the study protocol (IRB approval number: EK335112009).

**Fig 1:**
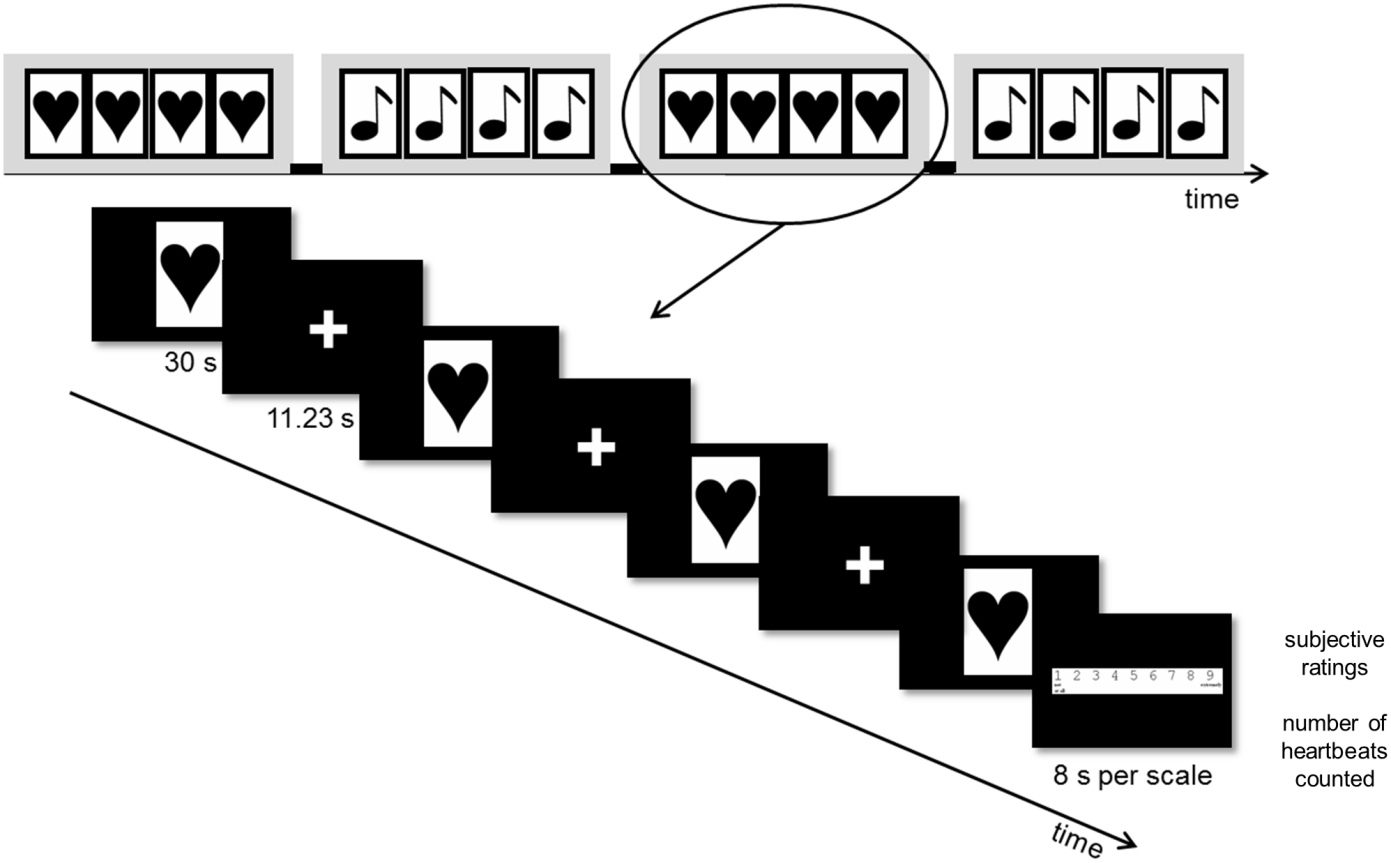
Design of the heartbeat paradigm. Upper half: Illustration of the block design structure (four conditions with four blocks of stimulation each, separated by 30 s baseline periods). Order of blocks was randomized across subjects. Lower half: Temporal sequence within one condition, using the example of the heartbeat condition. Hearts illustrate the heartbeat condition; clefs illustrate the sinus tone condition.

The heartbeat task exposed subjects to auditory heartbeat sounds in order to investigate the reactivity towards cues of interoceptive relevance. In a 2 x 2 factorial design, stimulus type (heartbeats vs. sinus tones) and frequency (resting vs. accelerated: 50 vs. 100 bpm) were manipulated, resulting in four experimental conditions presented in randomized order across subjects: resting heartbeat, accelerated heartbeat, resting tone, accelerated tone (see Fig 1 for details). Cardiac stimuli consisted of computerized heartbeats (acquired from www.soundsnap.com) that were looped in the given frequency for 30 s blocks, whereas the non-cardiac control stimuli were composed of sinus tones that were matched for frequency and loudness. Further, we induced a beat-to-beat variability in both heartbeats and sinus tones on a 0.1 Hz frequency band to simulate heart rate variability (Sounds available from the authors upon request.). Heartbeat and tone stimuli were presented in sufficient sound intensity to enable accurate stimulus discrimination from gradient background noise. Indicated by a visual cue (heart vs. clef), a block comprised the presentation of one condition for 30 s, repeated four times, separated by a fixation cross (inter-stimulus interval: 11.23 s) and followed by an inter-trial interval period of 30 s that served as baseline (Fig 1). Subjects were instructed to listen attentively to the auditory stimuli and to count heartbeats or tones in order to keep attention processes comparable between conditions. Further, subjects were instructed to imagine that they were listening to their own heartbeat in the heartbeat conditions. After each block, subjects completed ratings on the valence dimension (“I feel good/ bad.”), arousal (“I am nervous.”), anxiety (“I feel anxious.”), and the authenticity of the heartbeat stimuli (heartbeat condition only) on a 9-point Likert-type scale (“not at all” to “extremely”; “negative” to “positive” for valence ratings) and reported the total number of heartbeats or tones counted. Total duration of the task was 14:58 min. The paradigm was programmed and presented in Presentation 11.3 (Neurobehavioural Systems, Albany, CA, USA).

### SC data acquisition and analysis

SC was recorded with MRI compatible Ag/AgCl electrodes (MES Medizintechnik, Munich, Germany) with isotonic electrode paste as contact medium (Synapse, Kustomer Kinetics, Arcadia, CA, USA). Electrodes were attached to the second phalanx of the second and third index finger of the non-dominant hand, approximately five minutes prior to fMRI assessment (adaptation phase). Recordings were carried out using Brain Vision hard- and software (Brain Vision ExG Amplifier and Brain Vision Recorder; Brain Products, Munich, Germany). Raw data was filtered using a low cut-off (10 s) and high cut-off (250 Hz) filter; initial sampling rate was 1000Hz, SC data were subsequently downsampled on 10 Hz. A decomposition analysis of SC data was carried out in Ledalab Version 3.06 [27], a Matlab based application. Within this block task design, parameters of tonic SC were analyzed, including the mean number (#NS.SCR) and sum amplitude (AMP.NS.SCR; response criterion 0.02 μS) of non-specific SC fluctuations for each experimental condition. All SC parameters were range-corrected according to the method introduced by Lykken [28].

### Statistical analyses on subjective and SC data

Task effects on subjective ratings and SC data were analyzed using two-factorial ANOVAs with the two within-subjects-factors stimulus type and frequency, with the exception of authenticity ratings which were available for the heartbeat condition only (one-factorial ANOVA). Pearson correlations were computed between estimated ß-values and behavioural measures indicating trait (ASI) and state (subjective arousal and AMP.NS.SCR during H100 presentation) arousal processes. Bonferroni-corrections were applied to correlations to account for multiple testing. A level of α < 0.05 indicated statistical significance. Analyses were conducted in PASW 17.0 (SPSS Statistics, Armonk, NY, USA).

### fMRI data acquisition and analysis

Imaging was performed on a 3-Tesla TrioTim MRI whole-body scanner (Siemens, Erlangen, Germany). Whole-Brain Blood Oxygen Level Dependent (BOLD) contrast functional images were acquired using a T2* weighted gradient echoplanar images (EPI) sequence (41 transversal slices, slice thickness = 3.0 mm, in-plane resolution = 3 x 3 mm, interleaved collection, no gap, echotime (TE) = 25 ms, repetition time (TR) = 2500 ms, flip angle = 80°, field of view (FOV) = 192 x 192 mm, matrix = 64 x 64 mm, 357 volumes). Slices were acquired using a tilted angle to reduce susceptibility artefacts in inferior brain areas [29]. The first four scans were discarded to account for T1 equilibration effects, leaving 353 volumes for analyses. For anatomical reference, high-resolution structural images were collected after the experiment using a Magnetization Prepared Rapid Gradient Echo Imaging (MPRAGE) sequence (176 sagittal slices, slice thickness = 1 mm, TE = 2.26 ms, TR = 1900 ms, flip angle = 9°, FOV = 256 x 256 mm, matrix = 256 x 256). A standard 12 channel head coil was applied. Non-magnetic video goggles and head phones (VisuaStim Digital and SereneSound, Resonance Technology Inc., Northridge, CA, USA) were used; subjective ratings were provided using an optical response keypad (LUMItouch, Photon Control Inc., Burnaby, BC, Canada).

fMRI data were analyzed using statistical parametric mapping software (SPM5; http://www.fil.ion.ucl.ac.uk/spm/software/spm5/) on a Matlab platform (Matlab 7.1; The Mathworks Inc., Natick, MA, USA). EPIs were realigned and unwarped to correct for movement artefacts and visually inspected to control for excessive movements exceeding one voxel over time series. Structural images and EPIs were coregistered, normalized to the Montreal Neurologic Institute (MNI; Quebec, Canada) anatomical space, and spatially smoothed applying a Gaussian kernel of 8 mm, full width half maximum. Applying the general linear model (GLM), data were modelled for the four regressors of interest (resting heartbeat (H50), resting tone (T50), accelerated heartbeat (H100), accelerated tone (T100)); inter-stimulus intervals and rating phases within the blocks as well as the six movement parameters of the rigid body transformation were modelled as additional regressors of no interest. Individual t-contrasts from the first level comparing each regressor of interest to baseline (BL) (contrasts: H50>BL, H100>BL, T50>BL, T100>BL) were introduced into a second-level random effects analysis, in a region of interest (ROI) approach (exploratory whole brain analyses are given in the supplement). A full factorial design with the factors “stimulus type” (heartbeats vs. sinus tones) and “frequency” (50 vs. 100 bpm) was computed to examine neural activation for the main effects of stimulus type and frequency as well as the interaction of stimulus type and frequency; additionally, post-hoc t-contrasts were calculated for effect determination.

Anatomical ROIs were defined based on hypotheses on key structures of the interoceptive and threat processing network as outlined in the introduction (bilateral insular cortices, inferior frontal operculum, inferior, middle and superior frontal gyri, the anterior cingulate cortex, supplementary motor area, inferior parietal cortex, and amygdala) and were generated using the SPM toolbox WFU Pickatlas Version 2.4 [30, 31]. Functionally and anatomically associated brain areas, e.g. the insular cortices and the inferior frontal operculum, were merged into a combined ROI mask. For ROI analyses, threshold significance was p < 0.05 corrected for multiple comparisons using the false discovery rate (FDR; [32]) with a minimum cluster size of 10 contiguous voxels. Due to their exploratory character, whole brain analyses (as reported in the S1 Table) were not corrected for multiple comparisons and results are reported for p < 0.001 and a minimum cluster size of 10 contiguous voxels. Anatomical regions were assigned to peak voxel coordinates using Anatomical Automatic Labelling (AAL; [33]) in SPM. For correlation analyses with behavioural data, estimated ß-values of peak voxels within the insular cortex (if significantly activated according to ROI analysis) reflecting the magnitude of neural activation were extracted from the individual 1^st^-level model. A psychophysiological interaction analysis (PPI; [34]) was performed to assess functional connectivity between the observed task activation (peak voxel) with brain areas supposedly involved in interoceptive and threat processing. Deconvolved signal time series were extracted (corrected for the effect of interest) for each subject from a 5 mm sphere centered at a peak voxel coordinate identified in the group ROI analysis for the contrast heart > tone (right middle frontal gyrus: MFG; x = 42, y = 39, z = 3). The PPI model included three regressors modelling the psychological variable of interest (main effect of task contrast), the physiological variable of interest (deconvolved time series from the peak voxel coordinate), and their interaction (psychophysiological interaction; PPI regressor). Individual t-contrasts for the PPI regressor were introduced into a second-level random effects analysis, using a one-sample t-test. PPI results are reported for p < 0.05 (FDR corrected) and a minimum cluster size of 10 contiguous voxels. A second PPI on functional connectivity associated with the right insular cluster with a more liberal threshold (p < 0.01 uncorrected, minimum cluster size: 10 voxels) was also performed.

## Results

### Sample characteristics, subjective and SC data

**Fig 2:**
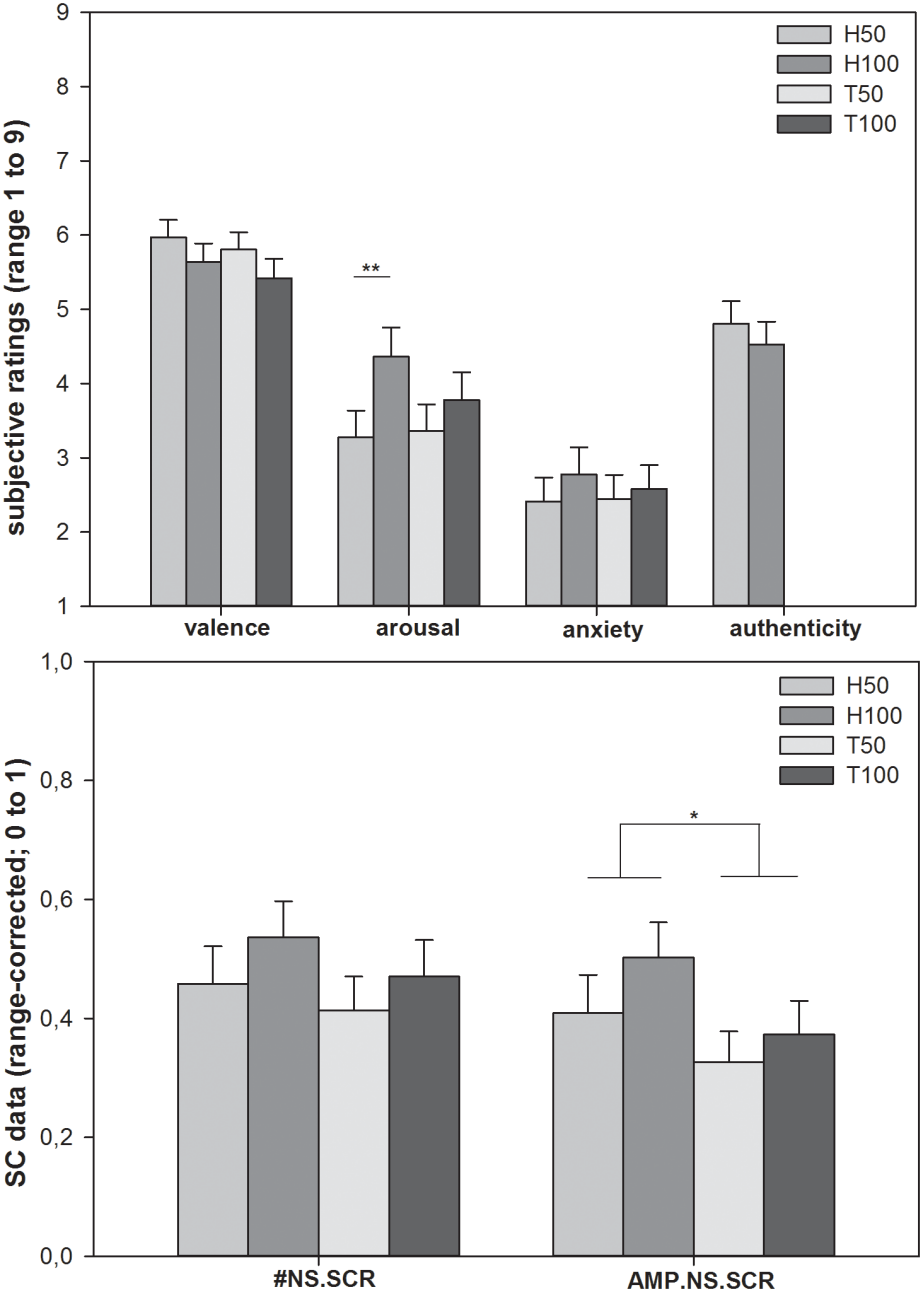
Behavioural data, illustrated separately for each experimental condition (H50, H100, T50, T100). Upper half: Subjective ratings on the dimensions arousal, valence, anxiety, and authenticity. Higher values indicate higher arousal, but positive valence. Lower half: Skin conductance data (range-corrected values). Error bars indicate the standard error of mean (SEM). #NS.SCR: mean number of non-stimulus specific skin conductance reactions; AMP.NS.SCR: mean amplitude of non-stimulus specific skin conductance reactions. * p < 0.05. ** p < 0.01

Demographics and questionnaire scores of the sample are depicted in Table 1. Subjective ratings are illustrated in Fig 2. For arousal ratings, significant results were obtained for the main effect of frequency and the interaction effect of stimulus type and frequency: Subjects reported significantly higher arousal after the accelerated heartbeat condition (arousal: interaction effect type x frequency: F (1, 35) = 7.778, p = 0.008; main effect frequency: F (1, 35) = 14.246, p = 0.001; main effect stimulus type: F (1, 35) = 1.255, p = 0.270). Regarding valence and anxiety ratings, no significant main or interaction effects were found, although a trend was observed for the main frequency effect on valence (valence: main effect frequency: F (1, 35) = 4.128, p = 0.050; main effect stimulus type: F (1, 35) = 1.283, p = 0.265; interaction effect type x frequency: F (1, 35) = 0.035, p = 0.852; anxiety: main effect frequency: F (1, 35) = 2.692, p = 0.110; main effect stimulus type: F (1, 35) = 0.376, p = 0.544; interaction effect type x frequency: F (1, 35) = 0.621, p = 0.436). With regard to the authenticity ratings, no significant differences emerged, i.e. the resting and accelerated heartbeat stimuli did not differ in terms of their perceived authenticity (authenticity: main effect frequency: F (1, 35) = 0.443, p = 0.510).

**Table 1:** Demographic characteristics and questionnaire scores of the sample. Means (SD) except where noted.

Sample; n = 36		
Female gender (n; %)	26	(72.2)
Smoking (n; %)	6	(16.7)
Age (years)	22.3	(3.1)
ASI (range 0–64)	16.4	(9.3)
BDI-II (range 0–63)	4.7	(5.4)

*Note*. Means and Standard Deviations are reported, except for the variables “Female gender” and “Smoking”. ASI = Anxiety Sensitivity Index; BDI II = Beck Depression Inventory II.

Analysis of tonic autonomic reactivity revealed higher amplitudes of SCRs during the heartbeat condition (AMP.NS.SCR: main effect stimulus type: F (1, 34) = 5.050, p = 0.031; AMP.NS.SCR: main effect frequency: F (1, 34) = 1.905, p = 0.177; interaction effect type x frequency: F (1, 34) = 0.180, p = 0.674; #NS.SCR: main effect frequency: F (1, 34) = 1.477, p = 0.233; main effect stimulus type: F (1, 34) = 1.588, p = 0.216; interaction effect type x frequency: F (1, 34) = 0.049, p = 0.827; see Fig 2 for details).

### fMRI Data

#### ROI analyses

Mirroring SC data, a significant main effect of stimulus type emerged: the post-hoc t-contrast heart > tone revealed significantly stronger activation towards the heartbeat stimuli in the right MFG, right inferior frontal operculum, bilateral insular cortices, left inferior frontal triangular gyrus, left superior MFG, and bilateral inferior parietal gyri. No other significant differences, neither for the main effect of frequency nor for the interaction effect of stimulus type and frequency, were found (Table 2). Further information on exploratory whole brain analyses is given in the S1 Table.

**Table 2:** Activation related to processing of heartbeat vs. sinus tones as revealed by a second-level random effects analysis (full factorial design with factors “stimulus type” and “frequency”), region-of-interest-analysis.

	Location	Side	MNI Coordinates (x, y, z)			F-/t-value	p corr.	cluster size
Main effect of stimulus type (F-contrast)	Middle frontal gyrus	R	42	39	3	24.92	0.003	167
	Inferior frontal operculum	R	42	15	33	17.22	0.023	86
	Inferior frontal gyrus triangularis	L	-42	24	6	16.75	0.010	13
	Middle frontal gyrus	R	45	3	54	14.41	0.015	10
t-contrast heart > tone	Middle frontal gyrus	R	42	39	3	4.49	0.002	242
	Inferior frontal operculum	R	42	15	33	4.15	0.011	147
	Inferior frontal gyrus triangularis	L	-42	24	6	4.09	0.005	87
	Middle frontal gyrus	R	45	3	54	3.80	0.008	45
	Inferior frontal gyrus triangularis	R	42	15	27	3.73	0.009	35
	Inferior parietal gyrus	R	42	-48	39	3.70	0.024	230
	Superior medial frontal gyrus	L	-9	30	45	3.50	0.013	10
	Insula^1^	L	-33	12	8	3.19	0.023	16
	Insula	R	39	15	3	3.11	0.025	11
	Inferior parietal gyrus	L	-47	-45	45	3.06	0.024	53
t-contrast tone > heart	*no significant activation*							
Main effect of frequency (F-contrast)	*no significant activation*							
Interaction effect of stimulus type and frequency (F-contrast)	*no significant activation*							

*Note*. p-values are corrected for multiple comparisons using the false discovery rate (p < 0.05). L = left; R = right; H50 = resting heartbeat; H100 = accelerated heartbeat; T50 = resting tone; T100 = accelerated tone; ^1^Peak voxel with 2.24 mm distance to the insula. Analyses are reported at p < 0.05 (FDR-corrected) with a minimum cluster size of 10 contiguous voxels.

#### PPI analysis

**Fig 3:**
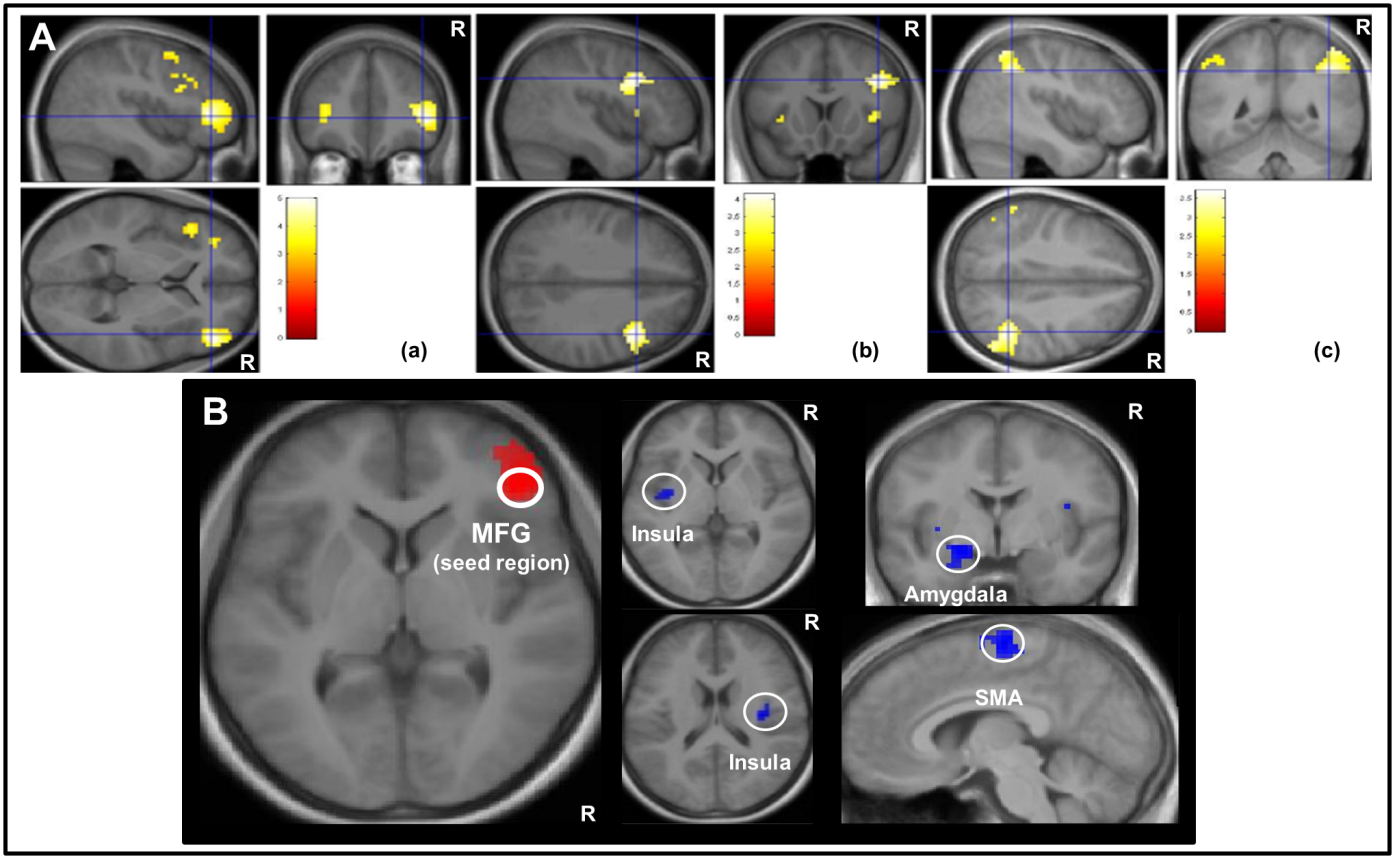
Functional brain activation and connectivity. A (upper line): Brain activation when listening to heartbeats in frontal (a), insular/ inferior frontal opercular (b) and parietal (c) regions as revealed by a second-level random effects analysis (full factorial design with factors “stimulus type” and “frequency”; contrast: H50+H100 > T50+T100), region-of-interest-analysis. Depicted brain activation is significant with p < 0.05 (FDR correction applied), cluster size > 10 contiguous voxels. a = Activation in the MFG and inferior frontal gyrus triangularis, crosshairs at (x = 42, y = 39, z = 3); b = Activation in the inferior frontal operculum and insula, crosshairs at x = 42, y = 15, z = 33); c = Activation in the inferior parietal gyrus, crosshairs at (x = 42, y = -48, z = 39). B (lower line): PPI analysis. The seed region in the MFG showed enhanced effective connectivity with the left and right insular cortices, the amygdala and the SMA. Brain activation is overlaid on an averaged high-resolution T1-weighted anatomical image of all n = 36 subjects, color-coded for t-values. R = right hemisphere.

The PPI analysis using the main task activation cluster in the right MFG revealed an enhanced functional connectivity with the bilateral insular cortices, the left amygdala and the supplementary motor area during the processing of heartbeat stimuli (contrast heart > tone; see Table 3, Fig 3 for details). Using the right insula as seed, an overlapping connectivity pattern was observed (Table 4).

**Table 3:** PPI results for the contrast heart > tone; seed region: middle frontal gyrus (x = 42, y = 39, z = 3).

Location	Side	MNI Coordinates (x, y, z)			t*-*value	p-value	cluster size
Supplementary motor area	R	6	-18	66	4.52	0.015	90
Insula	R	36	-15	18	4.44	0.030	48
Amygdala	L	-21	-3	-12	3.95	0.009	52
Insula	L	-36	-9	3	3.81	0.030	36

*Note*. p-values are corrected for multiple comparisons using the false discovery rate (p < 0.05). L = left; R = right; H50 = resting heartbeat; H100 = accelerated heartbeat; T50 = resting tone; T100 = accelerated tone.

**Table 4:** PPI results for the contrast heart > tone; seed region: right insula (x = 39, y = 15, z = 3).

Location	Side	MNI Coordinates (x, y, z)			t*-*value	p-value	cluster size
Amygdala	L	-21	-6	-12	3.95	< 0.01	12
Superior frontal gyrus	L	-24	39	45	3.41	< 0.01	13
Middle frontal gyrus	L	-36	15	39	3.19	<0.01	74

*Note*. Significance threshold: p < 0.01 (uncorrected); minimum cluster size: 10 voxels.

#### Correlations of fMRI data with subjective and SC data

**Fig 4:**
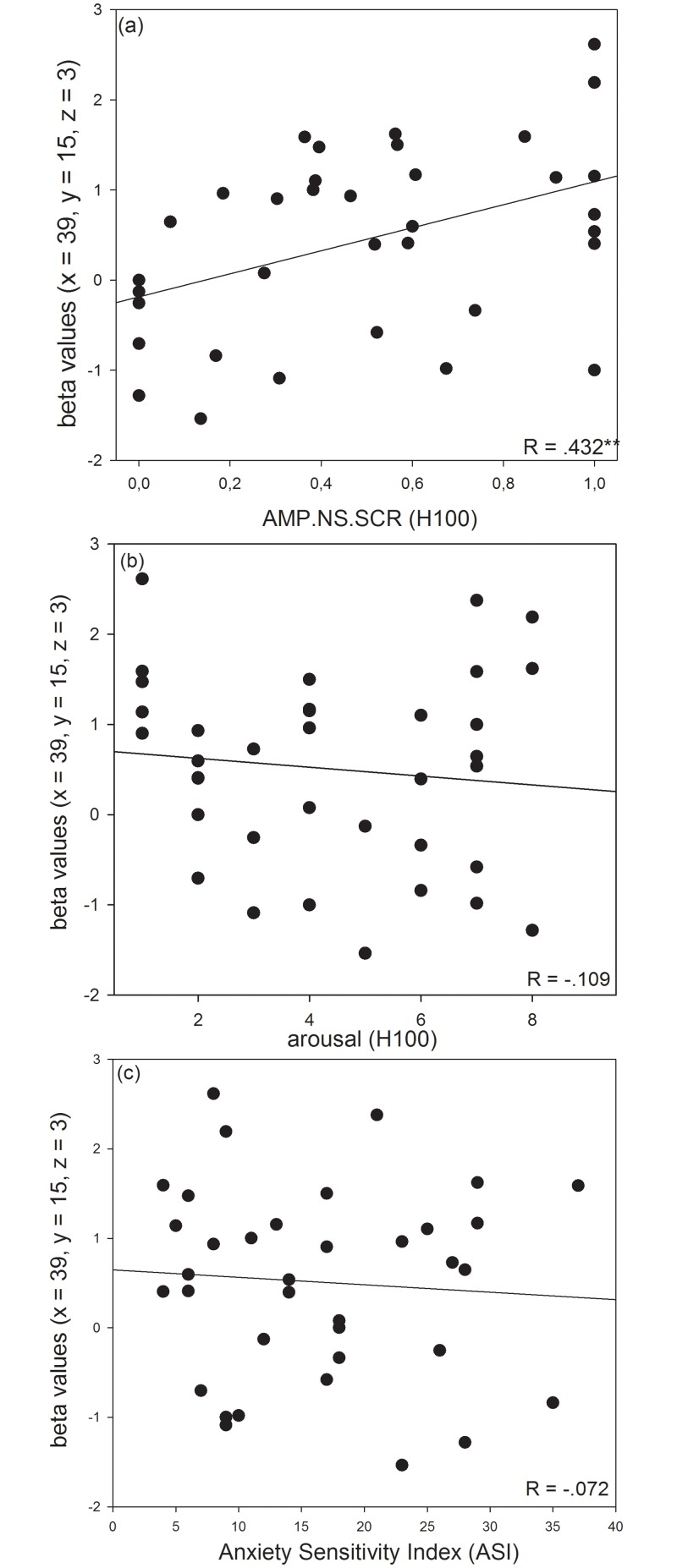
Correlation analyses. Scatterplots display correlations between the estimated beta values in the right anterior insular cortex (MNI coordinates (x = 39, y = 15, z = 3)) and AMP.NS.SCR (range-corrected) in response to H100 (a), subjective arousal ratings for H100 (b), and the Anxiety Sensitivity Index (c). Pearson correlation coefficients (R) are given in the plots. Estimated ß-values for the four regressors of interest (H50, H100, T50, T100) were extracted and added so that the t-contrast H50+H100 > T50+T100 was replicated, i.e. difference values were entered into correlation analyses. ** p < 0.01 (Bonferroni-corrected).

Estimated β-values for the peak voxel within the right anterior insular cortex correlated positively with SC amplitudes during stimulation with H100 (r = 0.432, p = 0.010; see Fig 4 for details), while no significant correlation with the MFG cluster was observed (r = 0.171, p = 0.325). Subjective arousal ratings subsequent to stimulation with H100 and the ASI total score were not correlated with insular activity (arousal: r = -0.109, p = 0.528; ASI: r = -0.072, p = 0.676).

## Discussion

Presentation of heartbeat sounds may initiate interoceptive processing, which, in turn, represents a core process for normal and pathological forms of emotional processing. Supplementing previous findings from heartbeat perception tasks (“Schandry-Task”), the present study yielded the following main findings: listening to heartbeat sounds (as compared to sinus tones) triggered activation in areas commonly associated with the processing of interoceptive information, including the bilateral insular cortices, inferior frontal operculum, and MFG. The magnitude of activation in one of the main interoceptive network nodes, e.g. the right anterior insular cortex, was positively associated with autonomic arousal. Main task activation in the MFG was positively coupled with activity in other key nodes of interoception and threat processing networks such as the insular cortex, the amygdala and the supplementary motor area. Results support the assumption that listening to heartbeat sounds induces subjective, psychophysiological, and neural processes linked to interoceptive information processing.

Using a multimodal assessment approach, behavioural data used in this study provide evidence that the task induced subjective and autonomic arousal associated with interoceptive processing. Subjects reported enhanced arousal following the heartbeat stimuli condition, particularly in response to the accelerated heartbeats, and displayed higher SC amplitudes during stimulation with heartbeat stimuli. In contrast, we did not observe effects on valence or anxiety ratings. As we investigated a healthy student population instead of anxiety-prone subjects or patients with anxiety disorders who may exhibit alterations in interoceptive processing [6, 23], it is conceivable that effects were predominantly observed in the arousal dimension, but were not perceived as threatening. In clinical populations, the present stimulus set may be perceived as more anxiety provoking, a hypothesis that should be tested in future studies.

Present neurofunctional data are in line with previous fMRI studies on the neural substrates of interoceptive processing. Emerging evidence suggests that the anterior insular and opercular cortices are related to attention to internal bodily sensations [35–37], with insular activation predicting accuracy during heartbeat monitoring [12] and local gray matter volume in the insula being associated with subjective awareness of visceral activation and interoceptive performance [11]. Present findings stress that merely listening to heartbeats already activates some parts of the interoceptive network as identified in previously used genuine interoceptive awareness tasks such as the “Schandry-Task”. Present findings also support previous studies, considering that enhanced bilateral insula and opercular activation were found during the heartbeat condition. However, in contrast to previous publications [11, 22] and theoretical considerations of neurobiological mechanisms mediating interoception [2], neither ACC nor PPC activation was identified in the present study. It has been suggested that the ACC is associated with second-order representations (or meta-representations) of the self [38], relevant for context evaluation and behaviour adjustment [23]. We hypothesize that, since subjects knew that the present stimulus was not their own heartbeat, it was not as relevant to their homeostatic state as genuine interoceptive information usually is in natural contexts; further, no changes in behaviour were required in response to the stimuli. Moreover, in contrast to Holtz et al. [16], the task did not include an anticipation phase so that anticipatory anxiety, with corresponding activity in the dorsal ACC, was unlikely to be observed. The inferior parietal activity identified in the present study can be further interpreted as a result of the continuous performance task: Subjects were instructed to count the number of stimuli; the left inferior parietal lobule is hypothesized to play an important role in number processing [39] and in the representation of quantity [40]. However, enhanced inferior parietal activation during the heartbeat condition compared to the tone control condition could indicate enhanced attentional resources during the heartbeat stimulus which is in line with findings of the inferior parietal lobule being associated with attention mechanisms, sensory processing, and sensorimotor integration [41]. Moreover, it could be hypothesized that differences in instruction, e.g. to imagine the heartbeat as one’s own, might have contributed to the differential activation pattern in the interoceptive network.

PPI results showed a functional interconnection between the main cluster in the heart > tone condition (MFG) and the interoceptive network and threat processing structures, particularly the bilateral insular cortices and the amygdala. The prefrontal cortex, which the MFG can be assigned to, is part of a neural system supporting emotion processing and regulation [42]. The PFC maintains reciprocal anatomical connections and functional interactions with the insula [43, 10], amygdala [44] and supplementary motor area. Within this framework, the insular cortex (alongside with other brain structures such as the amygdala) is involved in the identification of the emotional significance and salience of a stimulus and receives regulatory input from prefrontal areas. PFC and insula maintain reciprocal anatomical connections [43, 10], providing a plausible substrate for the functional coupling that was observed during listening to heartbeat sounds. Given that PPI analyses provide a measure of functional connectivity from which inferences on the causality of the identified relationships cannot be drawn [34], the direction of effects should nevertheless be interpreted with caution, even though a regulatory influence of the MFG on the insula appears to be in line with previous studies. The functional coupling of the MFG and amygdala further underlines the importance of previously reported prefrontal-amygdala-interactions involved in processing stimuli of emotional valence and biological significance [44]. Results using the insula as a seed yielded comparable results, albeit on a more liberal threshold (as the insula was not the main task activation cluster).

The association of insula reactivity with SC amplitude supports the role of the insula in autonomic arousal [45] which has also been identified in previous symptom provocation studies in clinical samples (e.g.[46, 47]). In contrast, no direct association was found with the MFG cluster, indicating that this structure may have a modulatory (see PPI results), yet indirect influence on the insula as a major interoceptive hub. The fact that insular reactivity towards heartbeats was not associated with either subjective arousal ratings or anxiety sensitivity could be attributed to the sample characteristics: We recruited a non-clinical sample of student volunteers that was not preselected according to any anxiety-related trait markers such as anxiety sensitivity. Anxiety-related state and trait markers may contribute to intra- and interindividual differences in interoceptive processing [6], but may only become manifest in high-risk or clinical populations, e.g. animal phobics [48]. For a better understanding of anxiety-related alterations regarding interoception, future studies should investigate changes during interoceptive processing in association with neural, subjective and autonomic markers using this task in anxiety-prone subjects.

Results demonstrate that already listening to heartbeats activated key structures of the interoceptive system. This approach can be regarded as a valuable alternative for studying interoception in experimental settings, particularly in fMRI studies, since scanner-specific setting properties (gradient background noise) may confound attention towards actual interoceptive stimuli. In particular, regarding heartbeat discrimination tasks, it has been argued that these tasks are rather difficult to accomplish, with untrained subjects not performing better than chance. With respect to fMRI this would imply that a considerable amount of subjects would not be able to perform this task successfully in the noisy scanner environment, making it difficult to interpret fMRI data. Compared to the high interindividual variance in perceiving one’s actual interoceptive state, the advantage of employing the current task is that it offers a comparable, continuous auditory stimulation across all subjects, enabling the experimental induction, manipulation, and measurement of interoceptive reactivity in a controllable setting. Nevertheless, it is noteworthy that the present paradigm did not involve an interoceptive task per se. No attentional switch towards internal, interoceptive stimuli was induced; instead, only exteroceptive stimuli were presented which might be reflected by the lack of activation in the ACC and PCC. It may be argued that genuine interoception could differ from listening to heartbeats in terms of implicated neural networks. As the present study showed substantial overlaps with results reported by other studies employing different paradigms for the assessment of interoception, we assume that attention to externally presented interoceptive cues vs. attention to internal bodily signs are both incorporated in the neurocircuitry of interoception (e.g. [2, 11]). Also, studies applying pharmacological manipulations [49, 50] revealed activity in brain regions such as the insula, the cingulum, and frontal areas being engaged in cardiovascular processing, thus providing further substantiation for the validity of the present task.

Limitations of the study should be taken into account for interpretation of results. First, we applied this task in a student population in order to assess basic neurofunctional correlates and to test the general feasibility, limiting the generalization to other populations such as anxiety-prone individuals or those with manifest disease. Second, the instruction to actually imagine that one is listening to one’s own heartbeat should have contributed to the perception of the heartbeats as authentic interoceptive stimuli, yet a differentiation of neural mechanisms involved in actual interoception is still to be explored. Third, the impact an external stimulation with heartbeat sounds could have on actual heartbeat should be investigated. Simultaneous electrocardiogram (ECG) recordings could be useful to examine heartbeat reactivity and whether an enhanced activation or deactivation in the heartbeat condition is a consequence of actual modification of the subject’s heartbeat; yet, methodological limitations refrained us from recording ECG signals online during scanning.

In conclusion, the present study showed that listening to heartbeats is accompanied by activation in brain areas associated with interoception and coupled to those neurofunctional systems subserving threat detection (e.g. amygdala). If replicated, this task could be applied to the investigation of pathophysiological mechanisms in pathological anxiety, where interoceptive dysfunctions are discussed to constitute a crucial component in the etiology and maintenance of anxiety-related psychopathology [6, 51, 52]. Due to its robust applicability in the fMRI environment, the present approach seems promising for studying neurobiological mechanisms and substrates of altered interoceptive processes in patient populations but also in subclinical, intermediate phenotypes such as anxiety sensitivity [23] to detect potential psychological vulnerabilities associated with altered interoception.

## Supporting Information

S1 Table Neurofunctional activation during the heartbeat task as revealed by a second-level random effects analysis (full factorial design with factors “stimulus type” and “frequency”), whole brain analysis. (DOC)
