# Supplementary material for: Probing the Interoceptive Network by Listening to Heartbeats: An fMRI Study
Source: PLoS One. 2015 Jul 23;10(7):e0133164. doi: 10.1371/journal.pone.0133164 (PMC4512728; doi:10.1371/journal.pone.0133164)
Supplement: S1 Table — (DOC) [file pone.0133164.s001.doc]

**S1 Table. Neurofunctional activation during the heartbeat task as revealed by a second-level random effects analysis (full factorial design with factors “stimulus type” and “frequency”), whole brain analysis.**

|  | Location | Side | MNI Coordinates  (x, y, z) | | | F-/t-value | p uncorr. | cluster size |
| --- | --- | --- | --- | --- | --- | --- | --- | --- |
| Main effect of stimulus type (F-contrast) | Middle frontal gyrus | R | 42 | 39 | 3 | 25.25 | <0.001 | 189 |
|  | Inferior frontal operculum^1^ | R | 36 | 0 | 27 | 18.26 | <0.001 | 90 |
|  | Inferior frontal gyrus triangularis | L | -42 | 24 | 6 | 17.04 | <0.001 | 13 |
|  | Superior parietal gyrus | R | 39 | -57 | 57 | 15.66 | <0.001 | 14 |
|  | Angular gyrus | R | 45 | -51 | 33 | 15.42 | <0.001 | 35 |
|  | Middle frontal gyrus | R | 45 | 3 | 54 | 14.44 | <0.001 | 12 |
|  | Supplementary motor area | R | 9 | 21 | 45 | 14.42 | <0.001 | 12 |
|  | Superior medial frontal gyrus | R | 6 | 33 | 39 | 13.96 | <0.001 | 14 |
|  | Cerebellum | L | -39 | -72 | -30 | 12.27 | <0.001 | 13 |
| t-contrast heart > tone | Middle frontal gyrus | R | 42 | 39 | 3 | 5.03 | <0.001 | 224 |
|  | Inferior frontal operculum^1^ | R | 36 | 0 | 27 | 4.27 | <0.001 | 137 |
|  | Inferior frontal gyrus triangularis | L | -42 | 24 | 6 | 4.13 | <0.001 | 25 |
|  | Superior medial frontal gyrus | R | 9 | 48 | 39 | 4.01 | <0.001 | 104 |
|  | Superior parietal gyrus | R | 39 | -57 | 57 | 3.96 | <0.001 | 104 |
|  | Caudate nucleus^3^ | R | 15 | -3 | 24 | 3.84 | <0.001 | 10 |
|  | Inferior frontal gyrus triangularis | L | -33 | 39 | 9 | 3.80 | <0.001 | 27 |
|  | Middle frontal gyrus | R | 45 | 3 | 54 | 3.80 | <0.001 | 28 |
|  | Cerebellum | L | -30 | -78 | -42 | 3.78 | <0.001 | 17 |
|  | Cerebellum | L | -39 | -72 | -30 | 3.73 | <0.001 | 36 |
|  |  |  |  |  |  |  |  |  |
| t-contrast tone > heart | *no significant activation* |  |  |  |  |  |  |  |
|  |  |  |  |  |  |  |  |  |
| Main effect of frequency (F-contrast) | Cerebellum | R | 21 | -60 | -21 | 20.11 | <0.001 | 57 |
|  | Precentral gyrus | L | -51 | -6 | 48 | 16.59 | <0.001 | 26 |
|  | Supplementary motor area | L | -3 | 3 | 60 | 14.19 | <0.001 | 10 |
|  | Precentral gyrus | L | -57 | 0 | 33 | 13.68 | <0.001 | 10 |
| t-contrast 100 > 50 | Cerebellum | R | 21 | -60 | -21 | 4.48 | <0.001 | 104 |
|  | Precentral gyrus | L | -51 | -6 | 48 | 4.07 | <0.001 | 68 |
|  | Supplementary motor area | L | -3 | 3 | 60 | 3.77 | <0.001 | 16 |
|  | Rolandic operculum | L | -48 | 6 | 3 | 3.51 | <0.001 | 27 |
|  |  |  |  |  |  |  |  |  |
| t-contrast 50 > 100 | Paracentral Lobule | L | -6 | -27 | 63 | 3.59 | <0.001 | 13 |
|  |  |  |  |  |  |  |  |  |
| Interaction effect of stimulus type and frequency (F-contrast) | Insula^2^ | L | -30 | -27 | 24 | 27.11 | <0.001 | 32 |
|  |  |  |  |  |  |  |  |  |
| t-contrast H50 > H100 | Paracentral Lobule | L | -9 | -21 | 66 | 3.53 | <0.001 | 19 |
|  |  |  |  |  |  |  |  |  |
| t-contrast H100 > H50 | Cerebellum | R | 21 | -60 | -24 | 3.86 | <0.001 | 11 |
|  |  |  |  |  |  |  |  |  |
| t-contrast T50 > T100 | *no significant activation* |  |  |  |  |  |  |  |
|  |  |  |  |  |  |  |  |  |
| t-contrast T100 > T50 | Insula^2^ | L | -30 | -27 | 24 | 4.26 | <0.001 | 42 |
|  | Fusiform gyrus | R | 33 | -75 | 0 | 4.06 | <0.001 | 12 |
|  | Precentral gyrus | L | -51 | -6 | 48 | 3.66 | <0.001 | 12 |
|  | Precentral gyrus | L | -54 | 0 | 33 | 3.36 | <0.001 | 15 |

*Note.* L = left; R = right; H50 = resting heartbeat; H100 = accelerated heartbeat; T50 = resting tone; T100 = accelerated tone; ^1^peak voxel with 2.24 mm distance to the inferior frontal operculum. ^2^Peak voxel with 2.24 mm distance to the insula. ^3^Peak voxel with 1.41 mm distance to the caudate nucleus. ^4^Peak voxel with 1.41 mm distance to the insula. ^5^Peak voxel with 5.48 mm distance to the insula. ^6^Peak voxel with 7.35 mm distance to the precentral gyrus. ^7^Peak voxel with 2.45 mm distance to the anterior cingulate cortex. ^8^Peak voxel with 3.00 mm distance to the pallidum. ^9^Peak voxel with 2.24 mm distance to the inferior parietal gyrus. Analyses are reported at p < 0.001 uncorrected with a minimum cluster size of 10 contiguous voxels.
